# Diathesis

**Published:** 1863-10

**Authors:** 

**Affiliations:** Illinois


DIATHESIS.—*By Dr. Anderson*, of Illinois.—[Being a Paper read at the last Meeting of the American Medical Association held at Chicago.]

The term diathesis is used to express any general constitutional condition which exerts modifying influences upon the course of disease.

These conditions produce very powerful and controlling effects upon the results of surgical injuries and operations, and to one of them alone, to-wit, the aplastic diathesis, ten per cent. of the deaths in surgical cases are due. This mortality is capable of being entirely prevented by means now within our knowledge and control. Yet the subject is difficult, and, being incapable of the attractions of pictorial illustration, is liable to be overlooked by superficial observers.

A great variety of diatheses exist, each having its own peculiar character; but the two which have the most important surgical relations, are the *aplastic* and the *hyperplastic.*

The *aplastic diathesis* is that condition of the system in which there is an excessive tendency to a dissolved condition of all protein compounds, the blood-corpuscles breaking down, the solid tissues readily ulcerating, and all the products of inflammation taking a liquid form, being either degenerated blood, serum, or pus. At the same time, there is a more or less striking absence of the power of depositing plastic lymph around inflamed points. It is in this diathesis alone that the patient becomes capable of those fatal aplastic diseases—traumatic erysipelas, diffusive phlebitis, pyæmia, and hospital gangrene. The deaths from these causes, amounting to ten per cent. of all mortality after surgical operations, may all be prevented.

The causes of the aplastic diathesis probably operate by inducing an excessively alkaline condition of the system. Alkalies are the natural solvents which in the human body maintain the liquid form of certain protein compounds, such as fibrin, albumen, caseine, etc., whether found in the blood, pus, or serum. It is probable, therefore, that an excess of these alkalies would have the effect to keep these compounds in the liquid form to an excessive extent. All the products of aplastic inflammation and effusion, whether blood, serum, or pus, are alkaline. Besides, the effluviæ of decomposing animal secretions, which are the most powerful external causes of aplasticity, are all saturated with alkaline gases of the ammoniacal series.

As was just remarked, the most powerful external cause of the aplastic diathesis is the exposure of the patient to the depressing alkaline effluviæ from decomposing pus, urine, or other animal products.

I saw this repeatedly exemplified during my service in the army. The crowding of too many wounded men into hospitals always produced within three days the evidences of aplasticity. Of two hospital steamers, after a battle near Vicksburg, where one was overcrowded and the other was not, the mortality on board the one not crowded was five per cent., and on the other, thirteen and a half per cent., the excess being due to erysipelas, pyæmia, and secondary hæmorrhages. So striking are these results that it is easy by the sixth day to distinguish the men who have lain in an overcrowded ward, simply by the appearance of the wounds. A thousand men of this sort, mixed with a thousand others who have been kept in perfectly pure air, could readily be separated by inspection of the wounds alone.

The effect of the aplastic diathesis is to prevent all that effusion of plastic lymph necessary to the repair of injured tissues, and to drain away the nutritive material of the blood in an excessive flow of pus or serum, thus exhausting the patient. In this diathesis incised wounds do not readily unite by first intention; lacerated wounds do not granulate freely; ulcerations become phagedænic; injured vessels reopen after ligature, and sound ones give way to ulceration, producing secondary hæmorrhage; and most important of all, the irritant animal poison found in erysipelas and hospital gangrene, when formed or received in any part of the body, spreads and produces rapidly fatal results, because its action is not limited by any barrier of plastic lymph.

The relation of this important poison to the aplastic diathesis is as follows:—The poison may be inoculated into a plastic constitution, but it will not there produce either erysipelas or hospital gangrene. The irritated spot is immediately surrounded by plastic lymph, a local abscess ensues, and the poison is expelled with the pus. A plastic constitution can not have erysipelas; but an aplastic one is liable to all the mischiefs resulting from diffusion of the poison in a liquid form through all parts of the body. I suppose that the suppurative inflammation of the internal coats of the veins results from the poisonous lymph taken up by the lymphatics at the affected part being carried into the venous current. Hence pyæmia.

The aplastic diathesis may exist without the poison, and the poison may be present without the diathesis; but when both are present, a fatal result is to be feared. Both the aplastic diatheses and the existence of the erysipelatous poison may be epidemic. The presence of these conditions produces a malignant character in the prevailing distemper. At such times malignant scarlet fever, hospital gangrene, puerperal peritonitis, confluent small-pox, and all other malignant local inflammations, are found to contain the erysipelatous poison, and are capable by inoculation or contagion of propagating erysipelas in aplastic, and abscesses in plastic constitutions.

The aplastic diathesis can be diagnosed in advance of a surgical operation, so as to enable the practitioner to guard against its effects. This may be done by carefully studying the condition of any abrasions, pimples, scratches, etc., some of which may be found upon the skin of almost every patient, or at any rate may be made in important cases for the purpose of diagnosis. They show the effects of the diathesis in the same manner as larger injuries.

The treatment of the aplastic diathesis consists—1st, In securing a perfectly fresh and pure air for respiration; 2d, the administration of such remedies internally and externally as will neutralize the alkalies. Such as the tincture of iron, iodine, chloride of zinc, sulphate of iron, bromines, sulphuric, muriatic, and nitric acids, etc. Chlorine, iodine, and bromine, not only neutralize alkalies, but destroy animal poisons. Practically, I use mur. tinct. of iron in doses of twenty drops internally every one or two hours, and tincture of iodine with glycerine kept constantly upon any local manifestation of the poison.

By the free use of the tincture of iron the diathesis may be changed from aplastic to plastic in thirty hours, and a marked improvement be manifested in the parts affected. For the past five years I have made a constant practice of giving muriated tincture of iron as a prophylactic after surgical operations, always commencing its administration in a few hours without waiting for any actual manifestation of aplastic disease. Since I have commenced this precaution, no patient of mine has ever died of traumatic erysipelas, phlebitis, or pyæmia, and yet I have operated in a vast number of cases, and ought, under the ordinary management, to have lost a number of patients by these complications. Erysipelas under this prophylactic treatment sometimes makes an effort to commence, but is readily conquered without dangerous results. I now feel perfectly safe in this respect, *and have ceased to reckon erysipelas, phlebitis, or pyœmia, among the risks of my operations*, if I can have control of the patient.

The *normal diathesis* is that where neither plasticity nor aplasticity is in excess, but where the medium happily prevails.

The *hyperplastic diathesis* is the opposite extreme from the aplastic. It is probably caused, as claimed by Fuller in his work on rheumatism, by the excess of acids in the system. It is marked by an excessive tendency to solid deposits in inflammation. Suppuration is difficult, and when it occurs, is surrounded by a hard plastic tumor. Wounds unite readily by first intention, but contusions and sprains form hard swellings, which are slow to suppurate, slow to resolve, and often keep the patient lamed for a year after the injury. Erysipelas, diffuse phlebitis, and pyæmia, are impossible, unless this diathesis is first overcome; but the innoculation of the erysipelatous poison results only in the formation of local inflamed tumors, which occasionally suppurate and discharge from the summit of a hard, well-defined swelling. Very painful felons occasionally result if the poison is applied to the hands.

The signs of this diathesis are:—1st, Any symptoms of genuine rheumatic tendencies—rheumatism being the typical disease of the hyperplastic diathesis as much as erysipelas is of the aplastic. 2d, Rapid drying up of scratches, abrasions, pimples, etc., upon the skin, without any tendency to suppuration. 3d, Absence of all disposition to pustular eruptions, the skin being clear, and often a little coarse, dry, and firm in its appearance. If there are any eruptions, they are apt to be of the scaly varieties, showing a tendency to excessive development of the cuticle.

I pass over the consideration of the cancerous, tuberculous, and syphilitic cachexies, for want of time at the present, designing to return to the topic at a future meeting.—*Amer. Med. Times.*

